# Hyperosmotic stress‐induced redistribution of pre‐mRNA cleavage factor I subunits is associated with shifts in alternative polyadenylation

**DOI:** 10.1002/2211-5463.70278

**Published:** 2026-06-04

**Authors:** Hitomi Soumiya, Masatake Osawa, Hidefumi Fukumitsu

**Affiliations:** ^1^ Laboratory of Molecular Biology, Department of Biofunctional Analysis Gifu Pharmaceutical University Japan; ^2^ Department of Molecular Design and Synthesis, Functional Biology Division Gifu University Graduate School of Medicine Japan

**Keywords:** alternative polyadenylation, CFIm25 (*NUDT21*), hyperosmotic stress, mammalian pre‐mRNA cleavage factor I (CFIm), stoichiometric stress response

## Abstract

Alternative polyadenylation (APA) is an important mechanism of cellular stress response mediated in part by the cleavage factor Im (CFIm) complex. However, the spatiotemporal dynamics and regulatory activity of the mammalian CFIm complex during stress remain poorly understood. In this study, we determined the effect of moderate hyperosmotic stress on CFIm localization and APA profiles in HEK293 cells. Using a dual‐normalization strategy that included *18S rRNA*‐ and a CDS‐based ratiometric qPCR, we identified a significant shift toward proximal polyadenylation sites (PAS) in the established CFIm targets, *NUDT21* (encoding CFIm25) and *DICER1*. Notably, these APA dynamics displayed distinct kinetic profiles influenced by the metabolic environment: While the *NUDT21* L‐3′UTR/CDS ratio recovered to baseline by Day 4, *DICER1* exhibited a serum‐dependent response, showing a progressive decline under low‐serum conditions but recovering under high‐serum conditions. Crucially, these alterations were absent in non‐target multi‐PAS genes such as *GOLGA2* and preceded any substantial reduction in total mRNA abundance, suggesting these effects represent a targeted regulatory event rather than a nonspecific byproduct of transcriptional decline. Mechanistically, hyperosmotic stress triggers a transient, coordinated redistribution of CFIm25 and CFIm68 from the nucleus to the cytoplasm, while total cellular protein concentrations remain stable. We propose that this spatial shift creates a ‘stoichiometric bottleneck’ within the nuclear CFIm pool, effectively limiting the processing of distal PAS. This ‘stoichiometric stress response’ offers a robust mechanistic framework linking subnuclear protein reorganization to the rapid reprogramming of the 3′UTR landscape, providing new insights into how cells modulate gene expression potential during osmotic adaptation.

## Graphical abstract

Hyperosmotic stress triggers the relocation of the CFIm complex from the nucleus to the cytoplasm. This shift creates a nuclear ‘stoichiometric bottleneck’, limiting CFIm availability for mRNA processing. Consequently, specific mRNAs like *NUDT21* and *DICER1* undergo targeted 3′UTR shortening, demonstrating how spatial protein dynamics drive rapid transcriptomic adaptation during environmental stress.

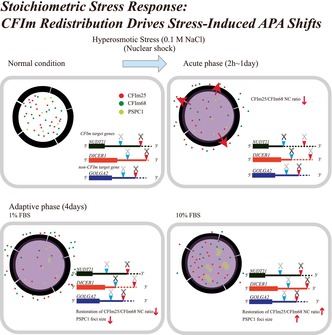

**Abbreviations**
APA: alternative polyadenylation
CFIm: mammalian pre‐mRNA cleavage factor I
HOPS: hyperosmotic phase separation
miRNA: microRNA
PAS: polyadenylation signals
PSPC1: paraspeckle component 1
UTR: untranslated region

## Alternative polyadenylation and its regulatory mechanisms

Eukaryotic mRNAs are processed by cleavage and polyadenylation at the 3′ end for stabilization. In the process known as alternative polyadenylation (APA), approximately 30–60% of the total mRNA contains multiple polyadenylation signals (PAS), one of which is exclusively selected for polyadenylation. Therefore, genes can produce a variety of mRNAs in the length of the 3′‐untranslated region (3′UTR) [1, 2]. Because the 3′UTRs are rich in specific binding sites for microRNAs (miRNAs) and RNA‐binding proteins, APA increases the diversity of post‐transcriptional regulatory mechanisms [3, 4].

## The role of CFIm in 3′ mRNA end processing

A core complex of approximately 20 different proteins regulates 3′ mRNA end processing [5, 6, 7]. Of these, mammalian pre‐mRNA cleavage factor I (CFIm) plays a key role in APA [8, 9]. CFIm25, which is encoded by *NUDT21* and also known as CPSF5, is a small subunit of CFIm. It binds to one of the large subunits (CFIm68, or 59 kDa; also known as CPSF6, and 7) to form heterodimers. Two mechanisms have been proposed for the formation of CFIm‐dependent long 3′UTRs. Either the CFIm complex contributes to the assembly of a stable 3′ mRNA end processing complex to the distal PAS, by binding to the UGUA enhancer at the ~ 55 nt upstream cleavage site [10], or when the CFIm binds two UGUA elements and forms heterotetramers to loop out the intervening RNA, which may select the distal PAS [11, 12].

## Stress‐induced APA shifts and subnuclear protein dynamics

Various stresses, including hyperosmotic stress and oxidative stress, trigger global 3′UTR shortening [13, 14, 15, 16]. This occurs through the preferential use of proximal PASs during stress conditions [13, 16], while recovery promotes the 3′UTR‐based degradation preferentially through long 3′UTR isoforms [13]. Shortened 3′UTRs enable transcripts to evade mRNA decay [4] and to repress miRNA‐mediated post‐transcriptional silencing [17, 18]. These cellular responses maintain homeostasis and facilitate cell survival during stress, whereas the mechanisms of global 3′UTR shortening are distinct among cell types and stress species and not fully understood.

Paraspeckle is a nuclear body that acts as a hub. It isolates and stores RNA‐binding proteins, including CFIm subunits, to modulate transcriptional and post‐transcriptional regulation [19, 20]. Paraspeckle formation is upregulated in response to diverse stressors [21, 22]. Although CFIm subunits are dispensable for paraspeckle formation [23], the CFIm68 subunit dynamically shuttles from the nucleoplasm to specific substructures, including paraspeckles [24]. Therefore, the molecular dynamics of CFIm subunits with paraspeckle are likely important to elucidate the mechanism of stress‐induced 3′UTR shortening.

Recent studies prompted us to examine the subnuclear dynamics and spatial reorganization of the CFIm complex under hyperosmotic stress, with a particular focus on two emerging regulatory paradigms: (1) Hyperosmotic phase separation (HOPS): Rapid hyperosmotic stress triggers HOPS, which is driven by changes in cell volume, macromolecular crowding, and hydration caused by hyperosmotic stress [14, 25]. CFIm68 is sequestered into these phase‐separated condensates, which may lead to defects in mRNA cleavage and polyadenylation, ultimately resulting in the global disruption of transcription termination [14]. (2) Subcellular redistribution of nuclear factors: Beyond intranuclear condensates, hyperosmotic stress induces the large‐scale redistribution of various nuclear components, including paraspeckle‐associated proteins, from the nucleus to the cytoplasm [26].

In this study, we determined the effect of NaCl‐induced hyperosmotic stress on the subcellular localization of the CFIm complex and its role in regulating alternative polyadenylation (APA). By focusing on a suite of functionally distinct targets, including *NUDT21* and *DICER1* (an established CFIm target), and *GOLGA2* (a non‐target multi‐PAS control), we determined how the spatial redistribution of CFIm subunits correlates with shifts in the 3′UTR landscape. To ensure high quantitative rigor, we integrated spatially resolved single‐cell imaging with a refined dual‐normalization qPCR strategy. This approach enabled us to decouple APA‐specific site selection from global transcriptional fluctuations. The results support a model in which the transient localized depletion of the nuclear CFIm pool creates a subnuclear stoichiometric imbalance, which may serve as a primary driver of APA shifts in specific target mRNAs. This ‘stoichiometric stress response’ offers a novel mechanistic perspective on how cells rapidly modulate gene expression through the spatial reorganization of the 3′‐end processing machinery and establish a link between subnuclear protein dynamics and transcriptomic adaptation during osmotic stress.

## Methods

### Cell culture and stress treatment

Parental HEK293 cells (Flp‐In™‐293 Cell Line, Cat# R75007; Thermo Fisher Scientific Inc., Waltham, MA, USA) were cultured in Dulbecco's modified Eagle's media [DMEM containing 6400 g·L^−1^ NaCl (110 mm), 4.5 g·L^−1^ glucose (25 mm) and L‐glutamine (Cat# 044‐29 765; FUJIFILM Wako Pure Chemical Corporation, Osaka, Japan)], and supplemented with 100 units/mL penicillin and 100 μg·mL^−1^ streptomycin (Meiji Seika Corporation, Tokyo, Japan), 1 mm sodium pyruvate (Cat#190‐14 881; FUJIFILM Wako Pure Chemical Co.), MEM nonessential amino acid solution (Cat#139‐15 651; FUJIFILM Wako Pure Chemical Co.), and 1 or 10% fetal bovine serum (FBS; Hyclone, Thermo Fisher Scientific) at 37 °C in a 5% CO_2_ atmosphere. For RT‐qPCR, western blot analysis, and MTT assays, 1.0 × 10^6^, 7.5 × 10^5^, and 5.0 × 10^3^ cells were seeded into 6.0, 3.5 cm dishes, and per well (96‐well plate), respectively. For hyperosmotic stress assays, we exposed HEK293 cells to culture medium containing 0.1 or 0.2 m NaCl for 2 h, 1 day, or 4 days.

### Real‐time quantitative PCR

Cells were lysed using TRIzol reagent. The isolated RNA was purified using a Direct‐zol RNA Miniprep Kit (Zymo Research Corporation, Irvine, CA, USA), and was treated with DNase I based on the manufacturer's instructions. For cDNA synthesis, 1 μg of total RNA was reverse‐transcribed using the ReverTra Ace qPCR RT Kit (for reaction volume, 20 μL; TOYOBO, Osaka, Japan). For real‐time qPCR analysis, 1 μL of cDNA was added to a 20‐μL reaction mixture. For probe‐based qPCR, PrimeTime gene expression Master mix, (Integrated DNA Technologies, Redwood, CA, USA); and for SYBR‐Green I‐based qPCR, we used the Thunderbird Next SYBR qPCR mix (TOYOBO, Oska, Japan). Quantitative PCR was done using the CFX96 Real‐Time PCR Detection System (Bio‐Rad, Hercules, CA, USA). The data were analyzed using cfx maestro™ Software ver 1.1 (Bio‐Rad).

To ensure quantitative rigor in assessing alternative polyadenylation (APA) dynamics, a dual‐normalization strategy was used as follows:

1. Global Abundance: Target mRNA levels (CDS) were normalized to *18S rRNA* levels to monitor overall transcript abundance.
2. APA Shift (Ratiometric Analysis): To quantify shifts in poly(A) site selection, the Distal/Total Ratio was calculated by normalizing the distal 3′‐UTR signal to the corresponding gene‐specific CDS signal (Total).

This approach effectively decouples APA‐specific regulatory events from global fluctuations in transcription or mRNA stability. The primers and probes used for experiments are listed in Table S1. The reproducibility of all the data was confirmed through at least two independent replicates.

### Western blot analysis

The expression levels of specific proteins were evaluated by western blotting, following previously established protocols [27, 28]. In brief, cultured cells were rinsed with 1× TBS (10 mm Tris/HCl, pH 7.4, 0.15 m NaCl) and subsequently treated with a lysis buffer consisting of 20 mm Tris/HCl (pH 7.4), 150 mm NaCl, 2 mm EDTA, 1% NP‐40, 0.1% SDS, 1% sodium deoxycholate, and a cocktail of inhibitors (10 μg·mL^−1^ each of aprotinin and leupeptin, 50 mm NaF, 1 mm Na_3_VO_4_, and 1 mm PMSF). Protein concentrations were adjusted to 1–10 μg per sample according to the specific target (refer to Table S2). The lysates were separated via SDS/PAGE on 10 or 14% polyacrylamide gels and then electroblotted onto polyvinylidene difluoride membranes (Pall Life Sciences, Washington, NY, USA). To prevent nonspecific binding, membranes were blocked for 1 h at room temperature with 5% skim milk (FUJI‐FILM Wako Pure Chemical Corporation) in a buffer containing 20 mm Tris/HCl (pH 7.4), 0.5 m NaCl, and 0.05% Tween‐20. This was followed by overnight incubation at 4 °C with primary antibodies diluted in the recommended blocking solution (Table S2). After washing, the membranes were treated with alkaline phosphatase‐conjugated secondary antibodies (anti‐mouse or anti‐rabbit IgG; Promega, Madison, WI, USA) for 1 h at room temperature. Protein signals were visualized using nitroblue tetrazolium and 5‐bromo‐4‐chloro‐3‐indoryl phosphate. For quantification, the intensity of the resulting bands was measured by densitometry using imagej software (NIH).

### Immunocytochemistry

For immunofluorescence, 1.0 × 10^4^ cells were seeded onto coverslips (10 mm diameter; Matsunami Glass, Osaka, Japan) coated with Cellmatrix type IP (Nitta gelatin Inc., Osaka, Japan) in a 24‐well plate. The cells were fixed on coverslips for 10 min by adding an equal volume of 4% (w/v) paraformaldehyde (PFA; Nacalai Tesque, Kyoto, Japan) in 0.1 m phosphate buffer (pH 7.4) (4% PFA solution) to the culture medium. They were postfixed for 10 min at room temperature (r.t.) with the same fixative solution. After washing the cells with PBS, they were permeabilized for 30 min at 37 °C with 0.3% (v/v) Triton X‐100 in 0.1 m Tris/HCl buffer (pH 7.4). Next, the cells were treated for 30 min at r.t. with PBS containing 2% (w/v) Block Ace (Dainippon Pharmaceutical, Osaka, Japan) to reduce nonspecific antibody binding, followed by incubation with primary antibody at 4 °C overnight. The primary antibodies included mouse anti‐CFIm25 (Cat# SAB1404890, 1 : 1000; Sigma‐Aldrich, St. Louis, MO, USA), rabbit anti‐CFIm68 (Cat# A301‐358A, 1 : 1000; Bethyl Laboratories Inc., Montgomery, TX, USA), and PSPC1 (Cat# HPA038904, 1 : 1000; Sigma‐Aldrich). After washing three times for 5 min in PBS, goat anti‐mouse and goat anti‐rabbit IgG conjugated with Alexa Fluor 488 and 546 were added, respectively (1 : 1000; Invitrogen Life Technologies, Carlsbad, CA) and incubated at 4 °C overnight. The samples were washed with PBS, counterstained with Hoechst 33342, and mounted in CC/Mount (Diagnostic BioSystems, Pleasanton, CA, USA).

Fluorescence images for initial screening were acquired using an all‐in‐one microscope (BZ‐X800; Keyence, Osaka, Japan). The area of Hoechst 33342‐positive nuclei and the integrated fluorescence intensity (integrated brightness) of CFIm25 and CFIm68 were quantified semiautomatically using the Hybrid Cell Count system (BZ‐X800 analyzer software; Keyence, Osaka, Japan).

For precise subcellular localization analysis, images were captured using a Zeiss LSM 900 confocal laser microscope (Carl Zeiss, Jena, Germany). To ensure a representative assessment of the cell population, fluorescence intensity shifts between the nuclear and cytoplasmic compartments were quantified using a 20× objective. All images were acquired and processed using identical Airyscan settings to ensure consistent and comparable fluorescence intensity measurements.

Quantitative image analysis was done using cellprofiler (version 4.2.8). The nuclei were segmented based on Hoechst 33342 staining using the *IdentifyPrimaryObjects* module, with a defined diameter range of 25–80 pixels. The cytoplasmic region was defined as a perinuclear ring, which was generated by expanding the nuclear mask with the *ExpandOrShrinkObjects* module. To account for cell shrinkage and morphological changes during osmotic stress, the expansion parameters were optimized as follows: a 3‐pixel expansion was applied to the acute stress experiments (0.1 and 0.2 m NaCl for 2 or 24 h), whereas a 5‐pixel expansion was used for long‐term experiments (0.1 m NaCl for 1 and 4 days), in which cell morphology remained relatively stable. The fluorescence intensities of the target proteins were quantified in both compartments using the *MeasureObjectIntensity* module. The nuclear‐to‐cytoplasmic (N/C) ratio was calculated for each cell as the mean nuclear intensity divided by the mean cytoplasmic intensity. For statistical evaluation of protein redistribution, the median N/C ratio of all the segmented cells within each imaging field was used as the representative value for that field. This approach ensures that the statistical unit (*n*) reflects independent imaging fields rather than individual cells, thus minimizing the effect of single‐cell outliers. Representative single‐cell images are provided for visualization purposes, but they were not treated as independent data points for statistical testing.

In contrast, single‐cell data were used to evaluate the quantitative relationship between CFIm25 and CFIm68 expression, as well as the N/C ratios. For this correlation analysis, the fluorescence intensities of both subunits were plotted for each cell to capture the protein–protein stoichiometric relationship and cell‐to‐cell heterogeneity across all conditions. By pooling individual cell data from multiple imaging fields, the robustness of subunit coregulation and the effect of hyperosmotic stress on the relative abundance of the CFIm complex components at a single‐cell resolution were evaluated.

To characterize the fine subnuclear distribution, high‐resolution representative images were acquired using the Airyscan 2 SR (Super‐Resolution) mode with a 63× oil immersion objective at a 3.0× zoom. This dual‐imaging approach enabled a robust statistical assessment of protein redistribution and a detailed characterization of the subnuclear architecture at a single‐cell level. Foci were quantified as punctate fluorescent structures within individual cells using cellprofiler. Automated thresholding (Otsu method) was applied and remained constant across all conditions. Objects smaller than 6 pixels were excluded to filter out background noise. The number of foci per nucleus was calculated for individual cells to assess the heterogeneity of the subnuclear dynamics across time points.

### Statistical analysis

Data are expressed as the mean ± standard deviation. All statistical analyses were done using graphpad prism 10 version 10.6.1 for Windows (GraphPad Software Inc., San Diego, CA, USA) following the Prism 10 Statistics Guide (https://www.graphpad.com/guides/prism/latest/statistics/index.htm). The details of the statistical analysis are provided in the figure legends. Briefly, for multiple comparison testing, Tukey's multiple comparison test was performed after a one‐ or two‐way analysis of variance. In all cases, statistical significance was determined using a 95% confidence interval; thus, *P* < 0.05 was considered statistically significant.

## Results

### Cell viability and translational activities are altered after hyperosmotic stress loading

To determine how the intensity and duration of hyperosmotic stress affect cell viability, HEK293 cells were cultured in DMEM containing 1% or 10% FBS (1% or 10% FBS‐DMEM) and treated with 0.1 and 0.2 m NaCl. The rationale for using low‐serum (1% FBS) was to decouple the specific regulatory effects of osmotic stress from the confounding influences of cell proliferation and exogenous growth factors. Because enhanced use of proximal PASs is closely linked to rapid cell proliferation [17, 18, 29], inducing a relatively quiescent state by restricting serum enabled us to determine whether the observed APA dynamics were a direct consequence of hyperosmotic signaling, rather than a secondary effect of altered growth rate.

**Fig. 1:**
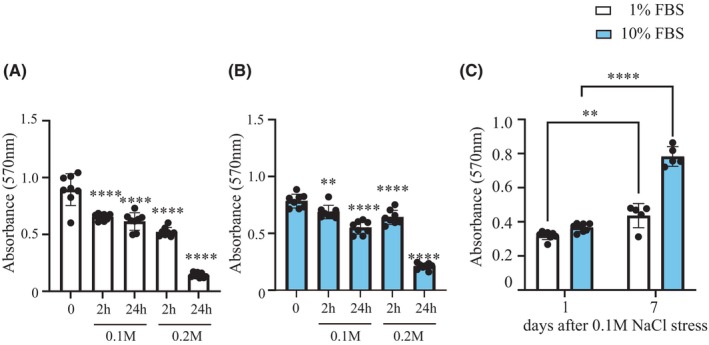
Time‐dependent cell viability under hyperosmotic stress: The viability of HEK293 cells was assessed using the MTT assay following 0.1 and 0.2 m NaCl treatment. HEK293 cells were seeded at a density of 5.0 × 10^3^/well. The cells were treated with the indicated concentrations of NaCl for the specified periods (A, B: 0.1 and 0.2 m for 2 and 24 h; C. 0.1 m for 1 and 7 days). In the MTT assay, the MTT reagent was added during the final 4 h of incubation. Following solubilization of the formazan crystals, the absorbance was measured at 570 nm. (A–C) Relative cell viability over time: The data are presented as the mean ± SD (A. B. *n* = 8; C. for *n* = 5  per group). Statistical significance was evaluated by a one‐way ANOVA followed by Dunnett's post hoc multiple comparison test. Significant differences compared with the untreated control (A, B: 0 h; C 1 day) are indicated (**P < 0.01, ****P < 0.0001).

Cell viability was measured by the MTT assay (Fig. 1). Under 0.1 m NaCl treatment, viability was significantly reduced at 2 h, regardless of serum concentration (1% FBS, 73.1%; 10% FBS, 87.8%), yet it remained relatively stable (1% FBS, 68.6%; 10%, 70.1%) (Fig. 1A,B). In contrast, 0.2 m NaCl treatment caused a marked, time‐dependent reduction in cell viability, dropping to 15.8% (1% FBS) and 26.9% (10% FBS) by 24 h (Fig. 1A,B). These results indicate that while 0.2 m NaCl‐induced severe cytotoxicity, the cells remained viable and capable of adapting to 0.1 m NaCl. Notably, MTT activity increased after 7 days of 0.1 m NaCl treatment under both serum conditions, which indicates that the cells remain proliferative under moderate osmotic stress, albeit at a reduced rate (Fig. 1C).

**Fig. 2:**
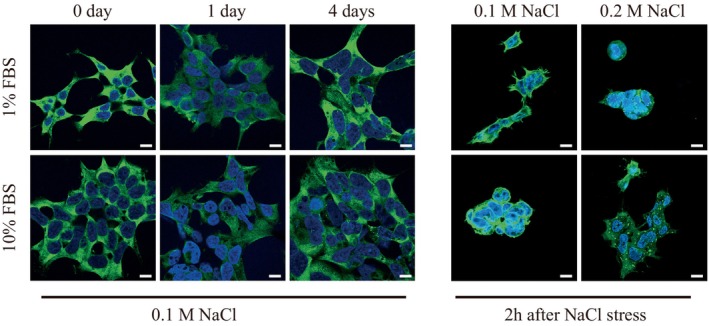
Hyperosmotic stress‐induced G3BP1‐positive stress granule formation. Representative immunofluorescence images of stress granule formation: HEK293 cells were cultured in 1% or 10% FBS‐DMEM and treated with NaCl as indicated. The cells were stained for the stress granule marker G3BP1 (green), and the nuclei were counterstained with Hoechst 33342 (blue). Time course images at 0, 1, and 4 days following 0.1 m NaCl treatment. Images captured 2 h after 0.2 m NaCl treatment are included as positive controls for the acute stress response. Scale bar = 10 μm.

To further characterize the cellular stress status, stress granule (SG) formation and translational activity were examined. At 2 h following 0.2 m NaCl treatment, G3BP1‐positive SGs were observed in most cells (Fig. 2, Table 1). G3BP1 is a nucleating factor for SGs [30, 31]. In contrast, SGs were scarcely detected at 2 h after 0.1 m NaCl treatment and remained undetectable through Day 4. Quantitative analysis indicated that the percentage of SG‐positive cells under 0.1 m NaCl did not significantly differ from untreated controls, whereas 0.2 m NaCl served as a robust positive control for SG induction.

**Table 1:** Quantitative analysis of G3BP1‐positive stress granule formation. The percentage of cells containing G3BP1‐positive stress granules was quantified under all experimental conditions. For each condition, 100–160 cells were analyzed from 7 to 10 randomly selected imaging fields. Data are presented as the mean ± SD from three independent experiments (*n* = 3). Statistical significance was determined using a one‐way ANOVA followed by Tukey's post hoc test for multiple comparisons. Significant differences compared with the control group were observed exclusively in the 0.2 m NaCl treatment group (****P < 0.0001 vs. untreated control).

	1% FBS‐DMEM	10% FBS‐DMEM
Control (0 day)	0	0
0.1 m NaCl (1 day)	0	0
0.1 m NaCl (4 days)	1.68 ± 0.97	1.94 ± 1.12
0.1 m NaCl (2 h)	0.93 ± 0.93	2.18 ± 1.50
0.2 m NaCl (2 h)	94.33 ± 0.87****	94.02 ± 2.20****

**Fig. 3:**
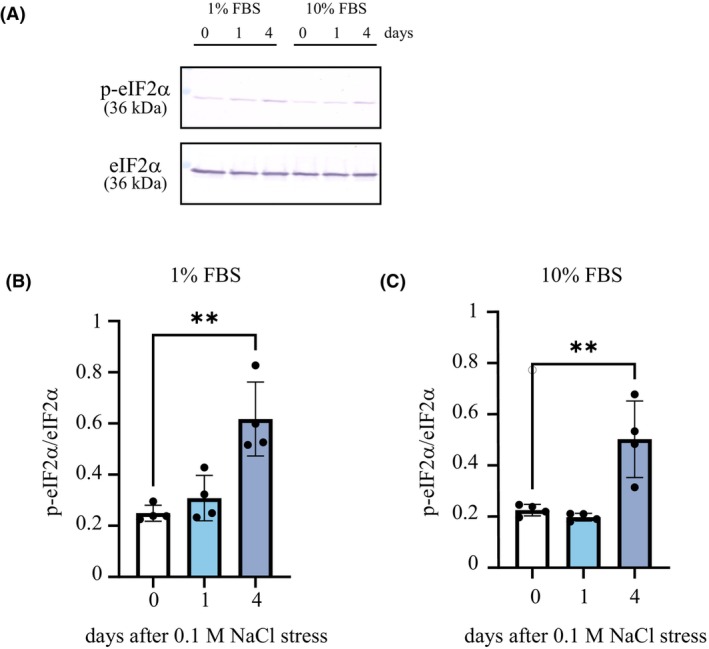
Effect of hyperosmotic stress on phosphorylated eIF2α proteins. (A). Representative western blot of eIF2α phosphorylation: HEK293 cells cultured in 1% or 10% FBS‐DMEM and treated with 0.1 m NaCl for the indicated times. Phosphorylated eIF2α (p‐eIFα) and total eIF2α levels were measured by western blot analysis. (B, C) Quantitative analysis of the p‐eIFα/total p‐eIFα ratio (B. 1%; C. 10%): Data are presented as the mean ± SD (*n* = 4 per group). Significant differences between groups were determined by a one‐way ANOVA followed by post hoc Dunnett's multiple comparison test (**P < 0.01).

Next, the phosphorylation of eIF2α, a key indicator of translational inhibition, was evaluated [32, 33]. Compared with untreated cells, eIF‐2α phosphorylation was significantly increased after 4 days of 0.1 m NaCl treatment, independent of serum concentration (Fig. 3, 1% 1d, *P* = 0.63, 4d, *P* < 0.005; 10% FBS‐DMEM, 1d, *P* = 0.87, 4d, *P* < 0.005).

This suggests that while moderate stress (0.1 m NaCl) does not trigger visible SGs, it significantly suppresses translational activity over time. Consistent with this, when 5.0 × 10^3^ cells/well were initially plated in 10% FBS‐DMEM under 0.1 m NaCl‐induced stress, the absorbance value increased only approximately threefold over 7 days (Fig. 1C). Because the theoretical doubling time of HEK293 cells is ~ 33 h, which would normally yield a ~ 16‐fold increase over 6 days, the observed proliferation rate under 0.1 m NaCl was markedly lower than expected, which reflects a sustained stress‐adaptive state.

### *NUDT21* 3´UTR shortening precedes the global impairment of transcriptional and translational activities under hyperosmotic stress

**Fig. 4:**
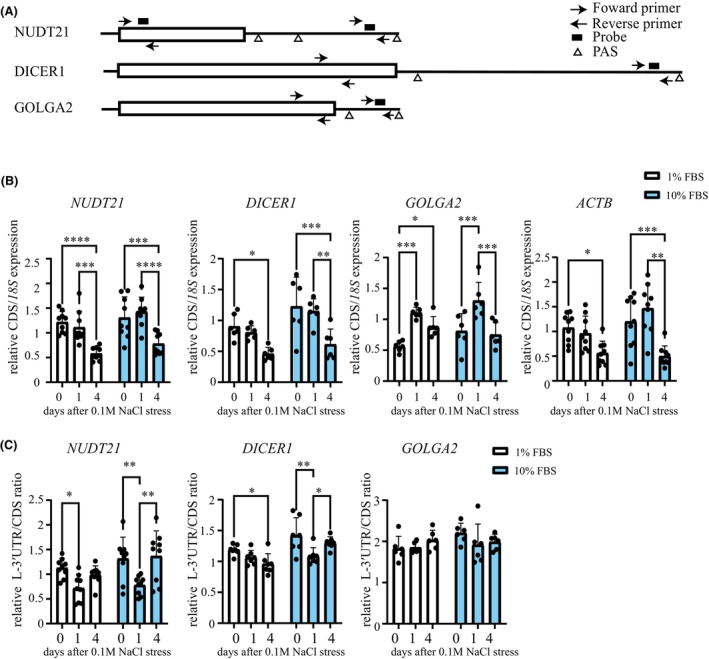
Hyperosmotic stress induces gene‐specific 3′UTR shortening. (A) Schematic representation of gene structures: Gene models for *NUDT21*, *DICER1*, and *GOLGA2* are shown. Open boxes indicate the coding sequence (CDS); horizontal lines represent the 5′ and 3′ untranslated regions (UTR); open triangles denote major polyadenylation sites (PAS); arrows indicate qPCR primer sets; thick bars represent probe recognition sites. (B) Effect of hyperosmotic stress and serum concentration on APA dynamics: HEK293 cells cultured in DMEM medium containing 1% or 10% FBS were treated with 0.1 m NaCl. At the indicated time points, the expression of the CDS and the distal 3′UTR regions was quantified by qPCR. Total mRNA levels were normalized to *18S rRNA* (CDS/*18S*). APA dynamics were evaluated by calculating the ratio of the distal 3′UTR to the CDS (L‐3′UTR/CDS), which represents the relative abundance of the long 3′UTR isoform. Bars represent the mean ± SD (*n* = 9 per group for *NUDT21* and *ACTB*; *n* = 6, or 7 per group for *DICER1* and *GOLGA2*). Significant differences between the groups were determined by a one‐way ANOVA followed by Tukey's post hoc multiple comparison test (*P < 0.05; **P < 0.01; ***P < 0.001; ****P < 0.0001).

The temporal effects of 0.1 m NaCl‐induced hyperosmotic stress on the expression and alternative polyadenylation (APA) of functionally distinct target genes, *NUDT21* (the autoregulatory core subunit), *DICER1* (an established CFIm target), and *GOLGA2* (a multi‐PAS non‐target control) were examined. Based on the PolyA_DB v4 database [34], the 3′UTRs of *NUDT21*, *DICER1*, and *GOLGA2* mRNA contain 3, 5, and 2 polyadenylation signals (PASs), respectively (Fig. 4A; [35, 36]). In the present study, we designated the 3′UTR isoform generated through the selection of the most distal PAS as the long 3′UTR (L‐3′UTR).

To distinguish between changes in overall transcript abundance and shifts in APA site selection, a dual‐normalization strategy was implemented. Specifically, total mRNA levels were quantified by targeting the coding sequence (CDS) normalized to *18S rRNA* (CDS/*18S*), which represents the overall transcript abundance. Simultaneously, the relative abundance of the distal PAS isoform was determined by calculating the ratio of the L‐3′UTR signal to the total CDS signal (L‐3′UTR/CDS), which provides a precise measure of APA site preference independent of total transcript fluctuations.

Compared with untreated cells, the total abundance (CDS/*18S*) of *NUDT21, DICER1*, and *ACTB* mRNAs remained stable at 1‐day post‐stress, but was significantly decreased by Day 4 under both 1 and 10% FBS conditions (Fig. 1B). For example, under 1% FBS conditions, *NUDT21* (1d: *P* = 0.71; 4d: *P* < 0.0001 vs. 0d) and *ACTB* (1d: *P* = 0.78; 4d: *P* < 0.005 vs. 0d) exhibited a delayed reduction in total abundance. The L‐3′UTR/CDS ratio, an indicator of APA preference, exhibited a markedly different kinetic profile (Fig. 1C). For *NUDT21*, the L‐3′UTR/CDS ratio was significantly decreased as early as 1‐day post‐stress, regardless of serum concentration. This APA shift recovered to baseline by Day 4 (1%: *P* < 0.05 at 1d, *P* = 0.62 at 4d vs. 0d, *P* = 0.20 at 4d vs. 1d; 10%: *P* < 0.01 at 1d; *P* = 0.94 at 4d vs. 0d, *P* = 0.001 at 4d vs. 1d). Notably, *DICER1* exhibited a serum‐dependent APA response. Under the 1% FBS condition, the *DICER1* L‐3′UTR/CDS ratio decreased progressively, reaching a significant reduction by Day 4 (1d: *P* = 0.32, 4d: *P* < 0.05 vs. 0d; 4d: *P* = 0.48 vs. 1d). In contrast, under the 10% FBS condition, *DICER1* showed a transient reduction followed by a successful recovery to basal levels by Day 4, mirroring the kinetics observed for *NUDT21* (1d: *P* < 0.005, 4d: *P* = 0.36 vs. 0d; 4d: *P* < 0.05 vs. 1d). Conversely, the CDS/*18S* levels of *GOLGA2* mRNAs were transiently increased at 1‐day poststress, but significantly decreased by Day 4 under 1% and 10% FBS conditions (1%: *P* < 0.0005 at 1d, *P* < 0.05 at 4d vs. 0d, *P* = 0.14 at 4d vs. 1d; 10%: *P* < 0.001 at 1d, *P* = 0.87 at 4d vs. 0d; *P* < 0.0005 at 4d vs. 1d). Importantly, these alterations were not observed in the L‐3′UTR/CDS ratio of *GOLGA2* (non‐CFIm targets) (1%: *P* = 0.99 at 1d; *P* = 0.44 at 4d vs. 0d, 10%: *P* = 0.21 at 1d, *P* = 0.41 at 4d vs. 0d), suggesting that early stage 3′UTR shortening is a gene‐specific regulatory event associated with the CFIm complex, rather than a nonspecific stress response common to all multi‐PAS genes.

To evaluate the technical validity of our normalization strategy, the behavior of *ACTB* was scrutinized. Although *ACTB* generates alternative 3′UTR isoforms in certain biological contexts [37], our preliminary analysis indicated that the *ACTB* CDS (CDS/*18S*, 1%: 2.62 ± 0.16 at 0d, 2.76 ± 0.34 at 1d, 1.36 ± 0.14 at 4d; 10%: 2.62 ± 0.54 at 0d, 3.6 ± 0.30 at 1d, 1.34 ± 0.56 at 4d, n.s. *n* = 3) and its distal 3′‐UTR (L‐3′UTR/*18S*, 5.28 ± 0.46 at 0d, 5.01 ± 1.35 at 1d, 2.56 ± 0.46 at 4d; 10%: 4.47 ± 1.1 at 0d, 5.72 ± 0.99 at 1d, 2.50 ± 0.34 at 4d, n.s. *n* = 3) displayed nearly identical expression kinetics throughout the stress time course. The absence of a significant shift in the L‐3′UTR/CDS ratio for *ACTB* (1%: 2.02 ± 0.10 at 0d, 1.83 ± 0.51 at 1d, 1.88 ± 0.16 at 4d; 10%: 1.72 ± 0.40 at 0d, 1.59 ± 0.14 at 1d, 2.02 ± 0.58 at 4d, n.s. *n* = 3), similar to our results for *GOLGA2*, supports the use of *ACTB* as a reliable reference for monitoring overall transcript abundance under these conditions.

**Fig. 5:**
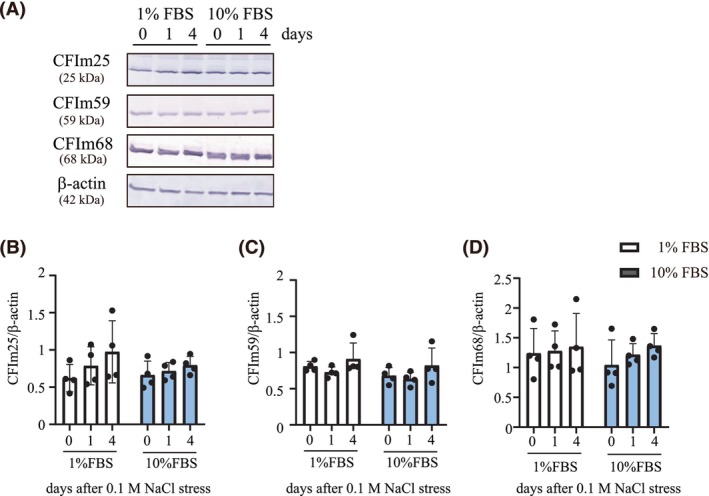
Hyperosmotic stress does not affect CFIm25, CFIm59, or CFIm68 protein expression. (A) Representative western blot analysis of the CFIm subunits: Expression of CFIm25, CFIm59, and CFIm68 was measured by western blot analysis using β‐actin as an internal control. (B–D) Quantitative analysis of CFIm protein expression: The protein expression levels of CFIm25 (B), CFIm59 (C), and CFIm68 (D) were normalized to β‐actin. The data are presented as the mean ± SD (*n* = 4 per group). Significant differences between groups were determined using a two‐way ANOVA followed by Tukey's post hoc multiple comparisons test.

Despite these transcriptomic shifts, the level of CFIm25, CFIm59, and CFIm68 protein remained stable over 4 days and showed no significant changes with either serum concentration (Fig. 5A–D). For example, CFIm25 protein levels remained constant in 1% FBS (*P* = 0.60 at 1d, *P* = 0.12 at 4d) and 10% FBS (*P* = 0.95 at 1d, *P* = 0.72 at 4d). These results suggest that the reduction in *L‐3′UTR/CDS* ratio for *NUDT21* and *DICER1* is a sensitive, early stage response that occurs while core protein levels are maintained.

These observations indicate that the 3′UTR shortening of CFIm targets represents an early stage molecular response that occurs independently of, and before, the broader decrease in global mRNA abundance. This temporal dissociation suggests that APA‐mediated regulation is a prioritized event and may act as an adaptive mechanism to modulate essential transcript isoforms before global cellular activities are compromised.

### Hyperosmotic stress triggers a sustained redistribution of CFIm subunits under lower serum conditions

Despite the significant shift in APA preference within 1 day, the protein levels of the key regulators, CFIm25 and CFIm68, remained unchanged (Fig. 5A–D). This discrepancy suggests that the functional impairment of the APA machinery is likely associated with the spatial redistribution of these factors, rather than a decrease in their total abundance. In HEK293 cells, CFIm68 is the predominant large subunit, and its depletion, unlike that of CFIm59, elicits a global effect on 3′UTR length [8, 9, 38]. Consequently, we focused on the CFIm68/25 axis as a potential factor in distal PAS selection for the remainder of the study.

**Fig. 6:**
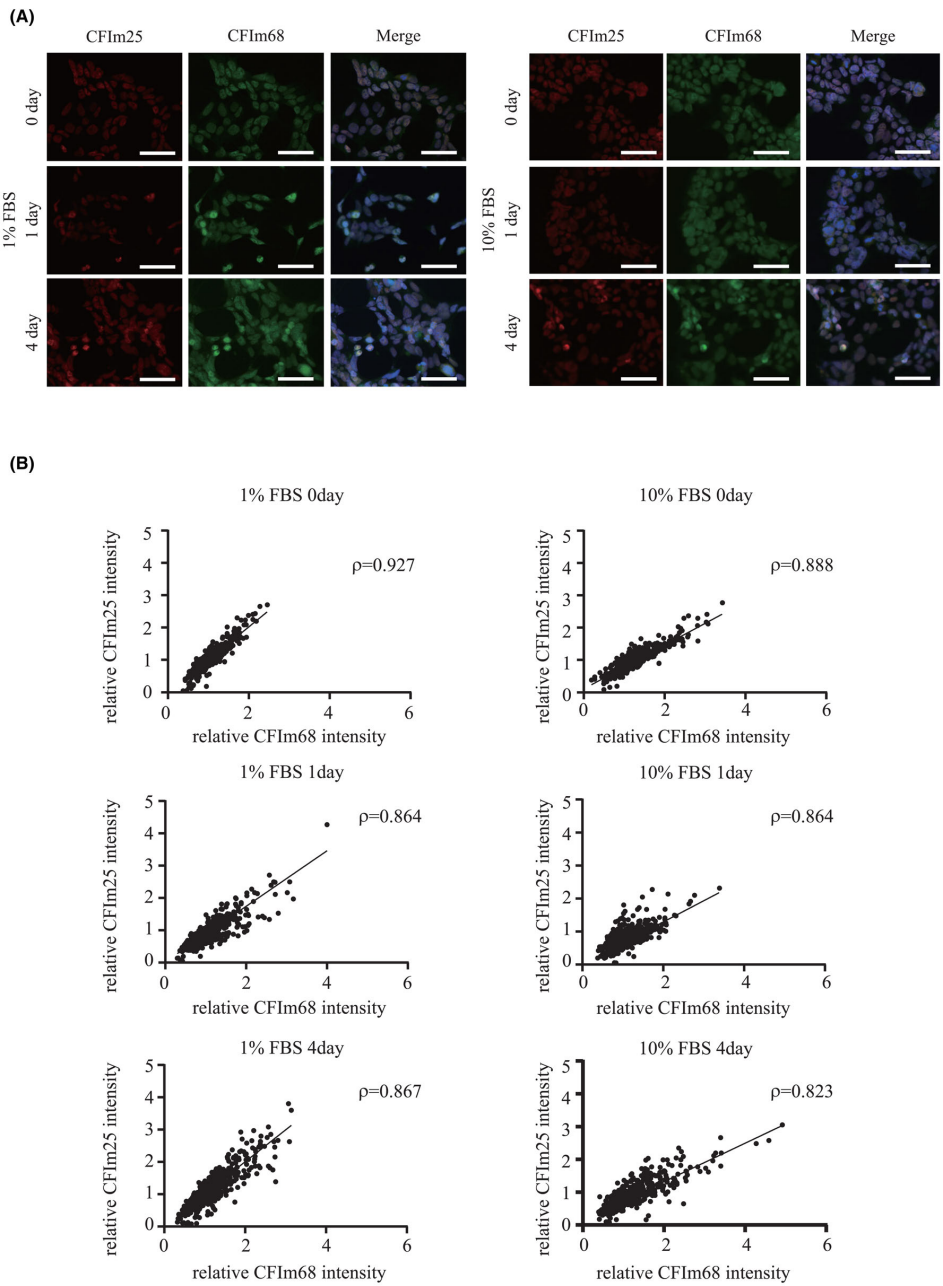
Robust coregulation of CFIm25 to CFIm68 expression under hyperosmotic stress. (A). Immunofluorescence imaging of the CFIm subunits: HEK293 cells were cultured in DMEM supplemented with 1% or 10% FBS. Following 0.1 m NaCl treatment for the indicated times, the cells were fixed and immunostained for CFIm25 (red) and CFIm68 (green). Nuclei were counterstained with Hoechst 33342 (blue). Scale bars = 50 μm. (B). Quantitative correlation analysis of CFIm25 and CFIm68 expression: Scatter plots illustrating the relationship between CFIm68 (*x*‐axis) and CFIm25 (*y*‐axis) fluorescence intensities in individual cells (*n* = 600 cells per group) under 1 and 10% serum conditions. The solid line indicates a robust regression fit used to minimize the effect of potential outliers. The correlation between the two subunits was determined by Spearman's rank correlation coefficient (*ρ*). Significant positive correlations were observed under all experimental conditions: 1% FBS: (Day 0: slope = 1.022, *ρ* = 0.927; Day 1: slope = 0.859, *ρ* = 0.864; Day 4: slope = 0.986, *ρ* = 0.867; all *P* < 0.0001). 10% FBS: (Day 0: slope = 0.8888, ρs = 0.672; Day 1: slope = 0.642, *ρ* = 0.864; Day 4: slope = 0.588, *ρ* = 0.823; all *P* < 0.0001)

Before assessing redistribution, the expression of CFIm25 and CFIm68 was evaluated at the single‐cell level. While the abundance of both subunits varied among individual cells (Fig. 6A,B), a strong positive correlation was maintained between CFIm25 and CFIm68 expression among all conditions (Fig. 6B). This suggests that the stoichiometry of the heterotetramer complex remains tightly regulated despite cell‐to‐cell variability. Notably, the ratio of CFIm25 to CFIm68 was not significantly altered by hyperosmotic stress, which confirms that the observed APA shifts were not caused by a stress‐induced imbalance in the total subunit pool.

Because hyperosmotic stress induces the large‐scale reorganization of nuclear components, a systematic time‐course analysis of CFIm localization was conducted using cellprofiler to quantify the nuclear‐to‐cytoplasmic (N/C) ratio. For robust statistical analysis, the median N/C ratio per imaging field was calculated, treating these as independent observations (*n*) to ensure that our statistical units reflect independent experimental measurements. To visualize the extent of cell‐to‐cell heterogeneity, N/C ratios for the individual cells were also plotted along with the aggregate data (Fig. S1).

**Fig. 7:**
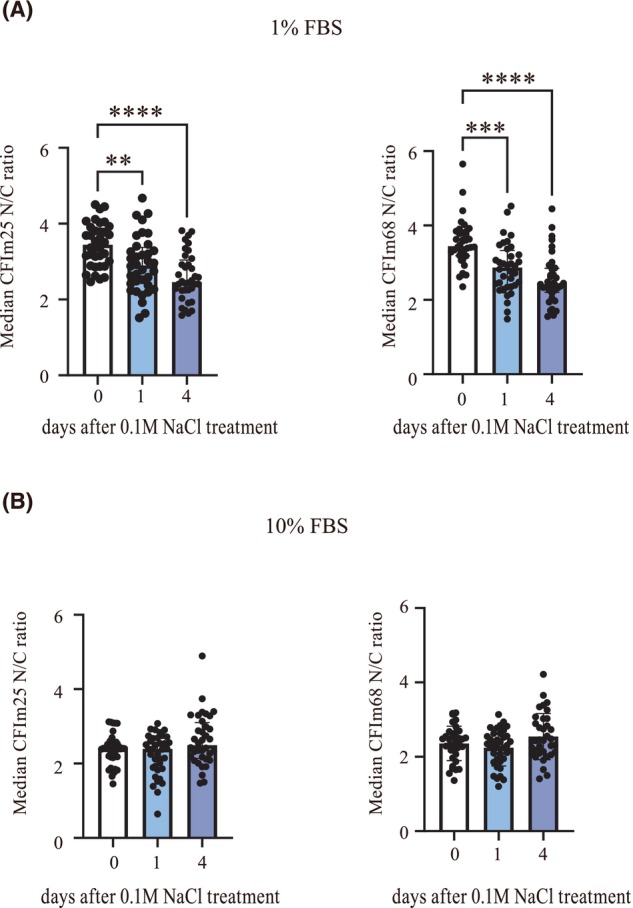
Hyperosmotic stress induces a sustained reduction in the nuclear occupancy of CFIm subunits under low‐serum conditions. HEK293 cells were cultured in 1% or 10% FBS‐DMEM and treated with 0.1 m NaCl for the indicated times. The cells were immunostained for CFIm25 (red) and CFIm68 (green), with the nuclei counterstained with Hoechst 33342 (blue). (A, B) Quantitative analysis of nuclear CFIm occupancy. The nuclear‐to‐cytoplasmic (N/C) ratio (representing nuclear occupancy) was quantified for CFIm25 and CFIm68. Each dot represents the median N/C ratio calculated from all segmented cells within a single imaging field (*n* = 36 fields per group; 6 fields per slide across 6 biological replicates). Horizontal bars and error bars indicate the group median and the interquartile range (IQR), respectively. (A) Under 1% serum conditions, the nuclear occupancy of both subunits was significantly decreased over the 4‐day stress period. (B) In contrast, under 10% serum conditions, the ratios remained stable at the daily intervals examined. Significant differences between groups were determined using a one‐way ANOVA followed by Tukey's post hoc multiple comparison test (**P < 0.01; ***P < 0.001; ****P < 0.0001).

Under 1% serum conditions, the overall nuclear occupancy of CFIm25 and CFIm68 was significantly decreased over the 4‐day stress period (Fig. 7A). Although these changes may not be immediately apparent upon qualitative inspection of individual subnuclear foci, our systematic quantification revealed a consistent reduction in the nuclear pool of CFIm subunits. In contrast, under 10% serum conditions, the N/C ratios for these subunits remained stable at the day level (Fig. 7B). Despite this stability, the N/C ratios of both subunits varied among individual cells, while maintaining a positive correlation among all conditions (Fig. S1). This suggests that the nuclear occupancy of CFIm25 and CFIm68 is coordinated at the single‐cell level, even when the overall N/C ratio is constant.

High‐resolution imaging indicated that CFIm25, CFIm68, and paraspeckle component 1 (PSPC1), a core paraspeckle protein, were predominantly localized within the nucleus as foci‐like structures (Fig. S2A–D). Interestingly, following hyperosmotic stress, cells with relatively larger PSPC1‐positive foci were occasionally observed, which appeared more frequently under the 10% serum conditions (Fig. S2D, arrowheads). Quantification of the foci exceeding a specific size threshold revealed divergent responses between the two conditions. Under 1% serum, the number of CFIm25‐ and PSPC1‐positive foci decreased, whereas under 10% serum, CFIm25 foci remained stable, while PSPC1 foci displayed an increasing trend (Fig. S3).

Although these qualitative observations suggest a link between subnuclear structural integrity and the retention of the nuclear CFIm pool, the day‐level distribution patterns do not fully account for the initial APA shifts of CFIm‐target genes, which occurred regardless of serum concentration.

### Acute‐phase redistribution of CFIm subunits and the divergence of recovery between stress intensities

To address whether the acute‐phase dynamics of nuclear molecules trigger the initial APA shifts, a high‐resolution analysis of CFIm localization at 2‐h poststress under 0.1 m and 0.2 m NaCl concentrations was performed.

**Fig. 8:**
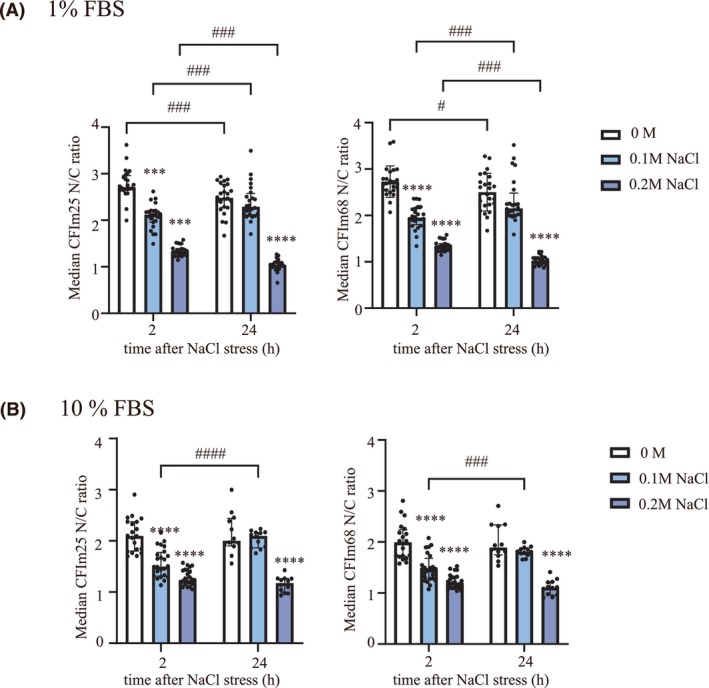
Differential dynamics of nuclear CFIm occupancy under moderate and severe hyperosmotic stress. HEK293 cells were cultured in DMEM containing 1% or 10% FBS and treated with either 0.1 m or 0.2 m NaCl for the indicated periods. Cells were immunostained for CFIm25 (red) and CFIm68 (green), with nuclei counterstained using Hoechst 33342 (blue). (A, B) Quantitative analysis of nuclear CFIm occupancy: The nuclear‐to‐cytoplasmic (N/C) ratio was quantified for CFIm25 and CFIm68 under 1% (A) and 10% (B) serum conditions. Under 0.1 m NaCl treatment, the nuclear occupancy of both subunits decreased transiently at 2 h, but rapidly recovered toward baseline levels by 24 h. This recovery was more robust under the 10% serum conditions. In contrast, under 0.2 m NaCl treatment, the reduction in nuclear occupancy was more pronounced and failed to recover within 24 h, regardless of serum concentration. Each data point represents the median N/C ratio calculated from all segmented cells within a single imaging field (*n* = 24 fields per group; 6 fields per slide across 4 biological replicates). Horizontal bars and error bars represent the group median and the interquartile range (IQR), respectively. Significant differences between groups were determined using the Kruskal–Wallis test followed by Dunn's post hoc multiple comparison test. Differences between concentrations at each time point (vs. 0 concentration) are indicated by * symbols, whereas differences over time within each concentration group are indicated by # symbols (comparisons shown in the graph) (**P < 0.01, ***P < 0.001; ****P < 0.0001, ^#^ *P* < 0.05, ^###^ *P* < 0.001, ^####^ *P* < 0.0001).

Our quantitative analysis revealed a significant, transient reduction in the nuclear CFIm pool, regardless of serum concentration at 2 h under 0.1 m NaCl stress (Fig. 8). In 10% serum, depletion was short‐lived and rapidly restored to baseline by 24 h (Day 1, Fig. 8). In contrast, under 1% serum, the initial recovery was weaker, eventually leading to the sustained reduction of the nuclear CFIm pool observed over the 4 days (Fig. 7). Notably, under the more severe 0.2 m NaCl stress, a more pronounced inhibitory effect was observed independent of serum concentration. Under these conditions, no recovery was observed, and the cells subsequently progressed toward cell death.

These results indicate that regardless of serum concentration, cells experience an immediate ‘nuclear shock’ and subsequent CFIm redistribution upon hyperosmotic stress. Only cells with sufficient serum support have the capacity to rapidly re‐establish the nuclear CFIm pool. This transient depletion at 2 h provides a possible mechanistic explanation for the initial APA shifts observed at Day 1 under both conditions.

High‐resolution imaging indicated that CFIm25 and CFIm68‐positive foci remained relatively evenly distributed within the nucleus at 2 or 24 h under 0.1 m NaCl (Fig. S4A–D, middle panels in A, B and upper panels in C, D); however, more severe osmotic stress (0.2 m NaCl) triggered a striking, time‐dependent nuclear‐to‐cytoplasmic translocation irrespective of serum levels (Fig. S4A–D, lowest panels). Under baseline or mild stress conditions, PSPC1‐positive foci were spatially distinct from the CFIm25‐positive foci (Fig. S5A–D, middle panels in A, B and upper panels in C, D). In contrast, under 0.2 m NaCl treatment, PSPC1‐positive foci were occasionally observed as larger, prominent spots before their eventual cytoplasmic translocation. By 24‐h post‐0.2 m stress, PSPC1‐positive signals were barely detectable within the cell nuclei (Fig. S5A–D, lowest panels).

These results indicate that hyperosmotic stress induces a spatial uncoupling of CFIm subunits and PSPC1, which disrupts the distribution and integrity of nuclear bodies.

## Discussion

In this study, we examined the spatiotemporal coordination between CFIm subunit dynamics and alternative polyadenylation (APA) to elucidate the mechanisms underlying 3′UTR shortening under hyperosmotic stress [14, 16]. Using a dual‐normalization strategy with 18S rRNA and CDS‐based ratiometric qPCR, we demonstrated that the Distal/Total (*L‐3′UTR/CDS*) ratio of established CFIm targets, *NUDT21* and *DICER1* [39, 40], significantly decreased 1‐day poststress (Fig. 4C). An important finding is the divergent recovery kinetics of these transcripts, which depend on the metabolic environment. For *NUDT21*, the APA shift returned to baseline by Day 4, a process independent of serum concentration, exhibiting a robust and autonomous recovery (Fig. 4C). In contrast, *DICER1* displayed a serum‐dependent response: while the *L‐3′UTR/CDS* ratio decreased progressively through Day 4 under 1% FBS, it showed a transient reduction followed by a successful recovery to basal levels under 10% FBS, mirroring the kinetics of *NUDT21* only when sufficient serum was available (Fig. 4C). Notably, the lack of such reduction in non‐CFIm targets (*GOLGA2* or *ACTB*) and the fact that APA shifts preceded substantial reductions in total mRNA abundance (Fig. 4) suggest that early stage 3′UTR shortening is a gene‐specific regulatory event rather than a nonspecific consequence of general stress or transcriptional decline.

Our data revealed a distinct temporal decoupling between APA shifts and global metabolic changes. Although increased eIF2α phosphorylation (Fig. 3, [32, 33]) and decreased *ACTB* mRNA (Fig. 4B) by Day 4 suggest late‐stage suppression of transcription and translation, the APA shifts were already prominent by Day 1. This highlights the role of APA as a rapid, early response system, distinct from the broader metabolic shifts that characterize the later stages of the stress response.

A central question is whether the nucleocytoplasmic redistribution of CFIm subunits and the subsequent APA shifts are functionally linked or represent independent stress‐induced events. High‐resolution imaging revealed that the N/C ratios of CFIm25 and CFIm68 decrease in a highly synchronized manner as early as 2‐h poststress, significantly preceding the manifestation of the APA shift (Fig. 8). This temporal precedence provides a rational basis for a causal link. Because CFIm25 serves as the essential scaffold for heterotetramer assembly [10, 11, 12], its transient nuclear depletion likely creates a ‘stoichiometric bottleneck’. According to the law of mass action, this reduction in the effective concentration of functional CFIm complexes would impair distal poly(A) site (PAS) processing. The observed positive correlation between the expression and N/C ratios of CFIm25 and CFIm68 (Fig. 6 and Figs S1, S6), even under stress, further supports the model that the availability of the heterotetramer complex is the primary determinant of target‐specific APA selection.

The divergent recovery kinetics between *NUDT21* and *DICER1* further refine this model, pointing toward an autoregulatory feedback loop [36]. Under 10% FBS, global nuclear localization of CFIm25/68 recovered by Day 1 (Figs 7 and 8), followed by the restoration of APA profiles by Day 4 (Fig. 4). In this context, *DICER1* behaves as a canonical downstream target, where its functional recovery is contingent upon the restoration of nuclear CFIm availability. In stark contrast, the recovery of *NUDT21* appears autonomous and decoupled from global protein re‐localization. Even under 1% FBS—where nuclear CFIm remains significantly reduced (Fig. 7)—the *NUDT21* APA profile returned to baseline (Fig. 4). This ‘extraordinary’ recovery suggests that *NUDT21* is uniquely sensitized to even trace amounts of nuclear CFIm or possesses a high‐priority ‘reset’ mechanism. As an essential component of the APA machinery, *NUDT21* likely prioritizes the restoration of its own distal PAS usage to maintain homeostatic control of the complex. We hypothesize that downstream targets like *DICER1* are subject to ‘transcriptional inertia’—a temporal lag reflecting the time required to turnover stress‐induced short isoforms—which requires serum‐derived metabolic support to overcome.

Furthermore, our results indicate that subnuclear organization does not fully return to homeostasis within 4 days. While hyperosmotic stress triggers hyperosmotic pressure phase separation (HOPS) in some models [14, 25], in our HEK293 model, CFIm subunits remained predominantly dispersed even under 0.2 m NaCl (Fig. S4). This suggests that nucleocytoplasmic translocation, rather than intranuclear sequestration, acts as the primary driver of stoichiometric imbalance. However, persistent alterations in PSPC1‐positive foci [41, 42] and their spatial dissociation from CFIm (Fig. S5) suggest that APA may be regulated by local subunit stoichiometry within specific subnuclear compartments rather than total nuclear abundance alone.

Finally, while the absence of direct rescue experiments remains a limitation, the spatiotemporal alignment and target specificity observed here strongly suggest that the repositioning of CFIm acts as a critical molecular switch. The fact that only CFIm‐target genes exhibit these shifts in correlation with CFIm redistribution argues against these being independent, coincidental events. Instead, this framework explains how subnuclear dynamics coordinate target‐specific transcriptomic adaptation. Future studies using optogenetic control of nuclear import will be essential to definitively establish the real‐time causality between subnuclear stoichiometry and PAS selection.

## Conclusion

We identified a novel ‘stoichiometric stress response’ mediated by the spatial redistribution of the CFIm complex and found that hyperosmotic stress triggers a coordinated reduction in nuclear CFIm25 and CFIm68 occupancy, which creates a functional bottleneck that favors proximal PAS selection. In addition, we revealed a compensatory recovery phase, in which the functional restoration of the APA landscape is decoupled from global protein relocalization, specifically in the autoregulatory *NUDT21* gene. These results reposition CFIm as a spatially regulated stress sensor and provide a new framework for how cells rapidly reprogram the transcriptome by modulating the subnuclear stoichiometry of the 3′‐end processing machinery.

## Conflict of interest

The authors declare no conflict of interest.

## Author contributions

HS contributed to investigation, formal analysis, methodology, visualization, writing – original draft preparation. MO contributed to writing – review and editing. HF contributed to conceptualization, data curation, formal analysis, funding acquisition, investigation, methodology, project administration, supervision, validation, visualization, writing – review and editing. We would also like to acknowledge members of the Fukumitsu laboratory for their careful reading of this manuscript.

## Supporting information


**Fig. S1.** Robust co‐regulation of nuclear CFIm25 and CFIm68 occupancy under hyperosmotic stress.
**Fig. S2.** High‐resolution analysis of CFIm and PSPC1 subnuclear localization.
**Fig. S3.** Quantification of large CFIm25 and PSPC1 foci.
**Fig. S4.** Effect of hyperosmotic stress on the subcellular localization of CFIm25 and CFIm68.
**Fig. S5.** High‐resolution analysis of CFIm25 and PSPC1 subcellular localization.
**Fig. S6.** Robust co‐regulation of nuclear CFIm25 and CFIm68 occupancy under modulate and sever hyperosmotic stress.
**Fig. S7.** Raw data for Western Blotting.
**Table S1.** Primer and probe sequences used for quantitative real‐time PCR analysis.
**Table S2.** List of antibodies used for western blot and immunocytochemistry in this study.

## Data Availability

The data that support the findings of this study are available in the Supporting Information of this article or from the corresponding author [hfukumi@gifu-pu.ac.jp] upon reasonable request.
