# Supplementary material for: Hyperosmotic stress‐induced redistribution of pre‐mRNA cleavage factor I subunits is associated with shifts in alternative polyadenylation
Source: FEBS Open Bio. 2026 Jun 4:10.1002/2211-5463.70278. Online ahead of print. doi: 10.1002/2211-5463.70278 (PMC13399382; doi:10.1002/2211-5463.70278)
Supplement: Supplementary file 1 — Fig. S1. Robust co‐regulation of nuclear CFIm25 and CFIm68 occupancy under hyperosmotic stress. Fig. S2. High‐resolution analysis of CFIm and PSPC1 subnuclear localization. Fig. S3. Quantification of large CFIm25 and PSPC1 foci. Fig. S4. Effect of hyperosmotic stress on the subcellular localization of CFIm25 and CFIm68. Fig. S5. High‐resolution analysis of CFIm25 and PSPC1 subcellular localization. Fig. S6. Robust co‐regulation of nuclear CFIm25 and CFIm68 occupancy under modulate and sever hyperosmotic stress. Fig. S7. Raw data for Western Blotting. Table S1. Primer and probe sequences used for quantitative real‐time PCR analysis. Table S2. List of antibodies used for western blot and immunocytochemistry in this study. [file FEB4-9999-0-s001.docx]

**
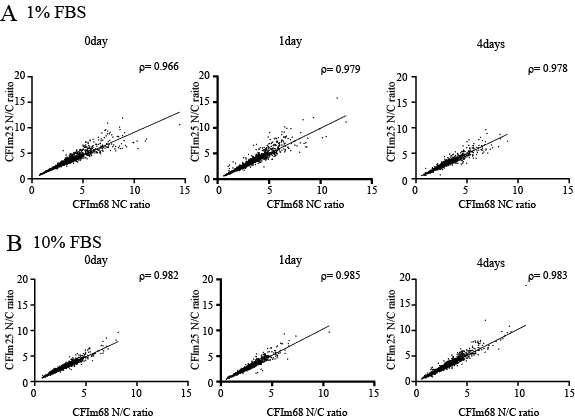
**

**Supplemental Fig. S1. Robust co-regulation of nuclear CFIm25 and CFIm68 occupancy under hyperosmotic stress.** A, B. Quantitative correlation analysis of nuclear CFIm occupancy: Scatter plots depicting the relationship between the nuclear occupancy (N/C ratio) of CFIm68 (x-axis) and CFIm25 (y-axis) in individual cells cultured under 1% (A) and 10% (B) serum conditions. To assess cell-to-cell variability in subnuclear distribution patterns, the N/C ratios of individual cells were pooled from all analyzed imaging fields (n = 1,400–2,300 cells per group). The solid lines represent a robust regression fit, used to minimize the effect of potential outliers. The correlation between the two subunits was determined by Spearman’s rank correlation coefficient. Significant positive correlations were observed across all experimental conditions:

- 1% FBS: (Day 0: slope = 1.022, ρ = 0.966; Day 1: slope = 0.859, ρ = 0.979; Day 4: slope = 0.978, ρ = 0.867; all *P* < 0.0001)
- 10% FBS: (Day 0: slope = 0.8888, ρs = 0.982; Day 1: slope = 0.642, ρ = 0.985; Day 4: slope = 0.588, ρ = 0.983; all *P* < 0.0001)

**
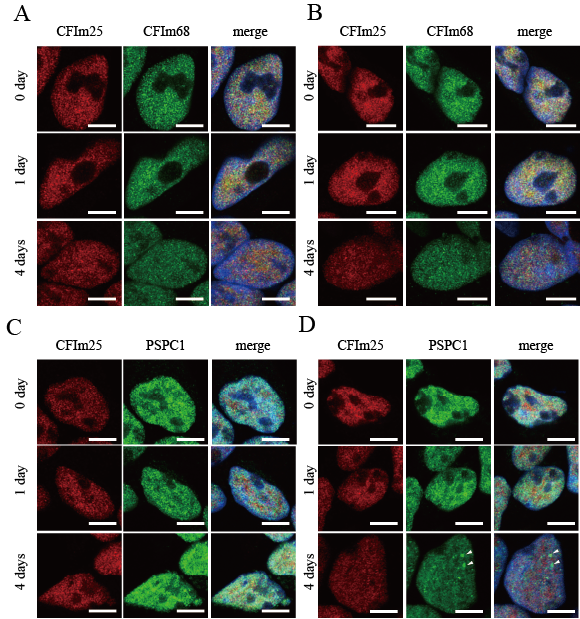
**

**Supplemental Fig. S2. High-resolution analysis of CFIm and PSPC1 subnuclear localization.** A–D. Representative magnified images of subnuclear foci: To visualize the fine internal structure of subnuclear foci, representative high-magnification images are shown. HEK293 cells were treated with 0.1 M NaCl-induced hyperosmotic stress for the indicated times in 1% (A, C) or 10% (B, D) FBS-DMEM. A, B: The cells were immunostained for CFIm25 (red) and CFIm68 (green). C, D. Subnuclear dynamics of CFIm and PSPC1: The cells were immunostained for CFIm25 (red) and PSPC1 (green). The nuclei were counterstained with Hoechst 33342 (blue) in all panels. Scale bars: 5 μm. Regardless of serum concentration, CFIm subunits-positive foci remained distributed throughout the nucleoplasm following 0.1 M NaCl treatment. In contrast, prolonged osmotic stress occasionally induced the formation of enlarged PSPC1 foci (D. arrowheads), which is a phenotype observed more frequently in cells cultured in 10% FBS-DMEM.


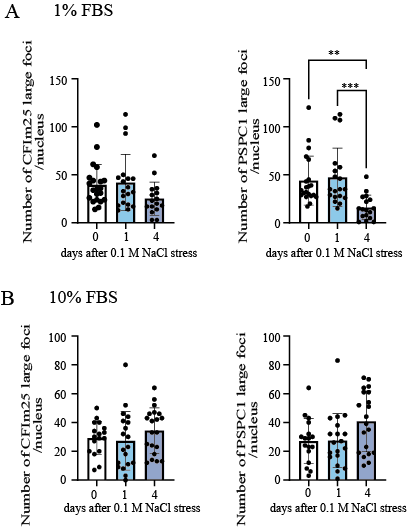


**Supplemental Fig. S3: Quantification of large CFIm25 and PSPC1 foci:** Analysis of subnuclear foci: The number of large subnuclear foci was quantified per cell using CellProfiler. Foci were defined as discrete, punctate fluorescent structures with an area >6 pixels. To ensure unbiased and reproducible object detection, automated Otsu thresholding was applied consistently for all conditions. This size-based filtering was used to focus on prominent protein clusters, while minimizing the influence of diffuse background signal and minor stochastic fluctuations. Dynamics of foci formation: Under 1% serum conditions, the number of CFIm25- and PSPC1-positive foci was significantly decreased by Day 4. In contrast, under 10% serum conditions, the number of CFIm25 foci remained stable, whereas PSPC1 foci showed a tendency to increase by Day 4. Statistical analysis: Data are presented as the mean ± SD (n = 17–21). Statistical significance was evaluated using Kruskal-Wallis test followed by Dunn’s post-hoc test for multiple comparisons. ***P* < 0.01; ****P* < 0.0001).

**
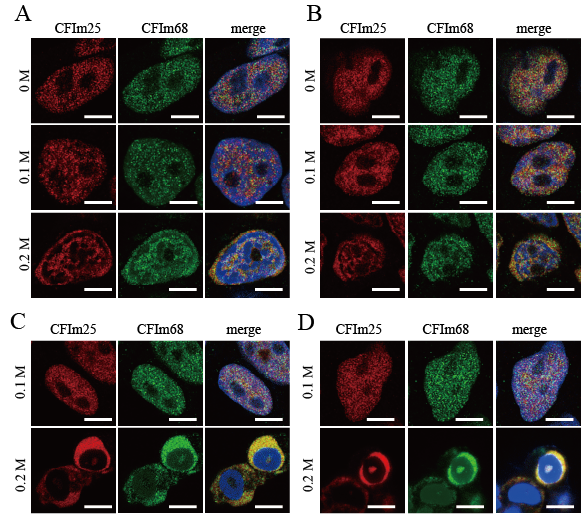
**

24h

2h

**Supplemental Fig. S4. Effect of hyperosmotic stress on the subcellular localization of CFIm25 and CFIm68.** HEK293 cells were exposed to hyperosmotic stress (0.1 and 0.2 M NaCl) for 2 h (A, B) or 24 h (C, D) in DMEM supplemented with 1% (A, C) or 10% (B, D) FBS. The cells were fixed and immunostained for CFIm25 (red) and CFIm68 (green), the nuclei were counterstained with Hoechst 33342 (blue). Under control conditions and 0.1 M NaCl treatment, CFIm subunit-positive foci were distributed throughout the nucleus regardless of serum concentration. In contrast, 0.2 M NaCl-induced hyperosmotic stress resulted in a time-dependent relocation of these foci to the cytoplasm; however, no distinct enlargement of the foci was evident. Scale bars: 5 μm.

**
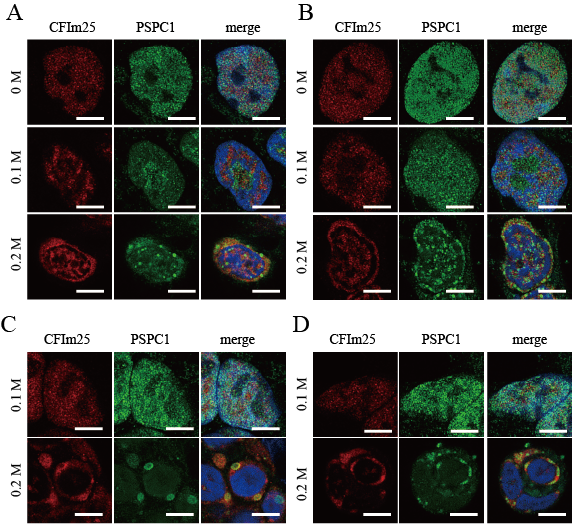
**

2h

24h

**Supplemental Fig. S5. High-resolution analysis of CFIm25 and PSPC1 subcellular localization.** A–D. Representative magnified images of CFIm25 and PSPC1 foci. To visualize the fine structural changes and subnuclear/cytoplasmic clustering, representative high-magnification images of single cells are presented. HEK293 cells were exposed to 0.1 and 0.2 M NaCl-induced hyperosmotic stress for 2 h (A, B) and 24 h (C, D) in 1% (A, C) or 10% (B, D) FBS-DMEM. The cells were immunostained for CFIm25 (red) and PSPC1 (green), with nuclei counterstained using Hoechst 33342 (blue). Scale bars: 5 μm. Detailed subcellular dynamics**:** Under control conditions and 0.1 M NaCl treatment, CFIm25- and PSPC1-positive foci exhibited distinct subnuclear distribution patterns; however, no significant changes in particle size or number were observed, regardless of serum concentration. In contrast, 0.2 M NaCl induced a time-dependent relocation of proteins to the cytoplasm, where they remained spatially distinct. Notably, for PSPC1, acute stage of severe stress (0.2 M NaCl) induced a morphological shift, characterized by an increase in particle size and a concomitant decrease in number, by 24 h, before any detectable cytoplasmic translocation.


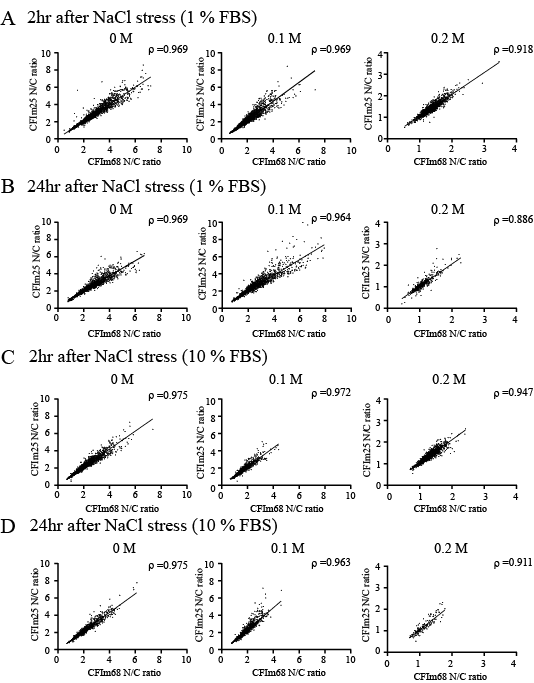


**Supplemental Fig. S6. Robust co-regulation of nuclear CFIm25 and CFIm68 occupancy under modulate and sever hyperosmotic stress.** A–D. Quantitative correlation analysis of nuclear CFIm occupancy: Scatter plots depicting the relationship between the nuclear occupancy (N/C ratio) of CFIm68 (x-axis) and CFIm25 (y-axis) in individual cells cultured under 1% (A, B) and 10% (C, D) serum conditions at 2 h (A, C) and 24 h (B, D) following NaCl-induced osmotic stress. To assess cell-to-cell variability in subnuclear distribution patterns, the N/C ratios of individual cells were pooled from all analyzed imaging fields (n= 139–440 cells, for 0.2 M at 24h; n = 1035–2,016 cells per other group). The solid lines represent a robust regression fit, used to minimize the effect of potential outliers. The correlation between the two subunits was determined by Spearman’s rank correlation coefficient. Significant positive correlations were observed across all experimental conditions:

- 1% FBS: (2h; 0M : slope = 0.99, ρ = 0.969; 0.1M : slope = 1.10, ρ = 0.969, 0.2M: slope = 1.08, ρ = 0.918, 24h; 0M: slope = 0.876, ρ = 0.969, 0.1M : slope = 0.906, ρ = 0.964, 0.2M: slope = 1.04, ρ = 0.886；all *P* < 0.0001)
- 10% FBS: (2h; 0M : slope = 1.05, ρ = 0.975, 0.1M : slope = 1.10, ρ = 0.947, 0.2M: slope = 1.08, ρ = 0.947, 24h; 0M: slope = 1.09, ρ = 0.975, 0.1M : slope = 1.28, ρ = 0.911, 0.2M: slope = 1.19, ρ = 0.911；all *P* < 0.0001).

**Supplemental Table S1 Primer and probe sequences used for quantitative real-time PCR analysis**

|  | Primer/Probe sequence | Accession No. | PCR Product size (bp) | Effici-ency(%) | R^2^ | Slope |
| --- | --- | --- | --- | --- | --- | --- |
| huACTB/CDS | Fwd: 5’-ACAGAGCCTCGCCTTTG-3’  Rev: 5’-CCTTGCACATGCCGGAG-3’ | NM_001101.5 | 110 | 99.9 | 0.998 | -3.33 |
| huACTB/L-3’UTR | Fwd: 5'-CGAGGACTTTGATTGCACATTG-3'  Rev: 5'-ACTGGGCCATTCTCCTTAGA-3'  Probe: 5'-/56-FAM/TTGTTACAG/ZEN/GAAGTCCC TTGCCATCC/31ABkFQ/-3' | NM_001101.5 | 124 | 105.0 | 0.993 | -3.21 |
| huDICER1/CDS | Fwd: 5'-CATGCCTCCTACCACTACAATAC-3'  Rev: 5'-GGTGCTTGGTTATGAGGTAGTC-3' | NM_001195573.1 | 94 | 89.2 | 0.999 | -3.46 |
| huDICER1/L-3’UTR | Fwd: 5’-GGAACATGATGTCATTCATTCATAC-3’  Rev: 5’-TTGGAGATTTACTTGGCTACAA-3’  Probe: 5'-/56-FAM/TGCTGCAGA/ZEN/AATTTGC AGTCTGCA/31ABkFQ/-3' | NM_001195573.1 | 88 | 94.7 | 0.986 | -3.42 |
| huGOLGA2/CDS | Fwd: 5'-CTGGAGAGACAGACACCATTG-3'  Rev: 5'-CCTTCATCTCCTCCTTGTCTTG-3' | NM_001366244.2 | 129 | 89.1 | 0.994 | -3.61 |
| huGOLGA2/L-3’UTR | Fwd: 5’-GCCTCCTTACTCTCACCAAAG-3’  Rev: 5’-CCTCGCCTGGTCTACAGTTA-3’  Probe: 5'-/56-FAM/CCCATCTTC/ZEN/TTACACA GAGAGGCAGC/31ABkFQ/-3' | NM_001366244.2 | 108 | 114.0 | 0.975 | -3.03 |
| huNUDT21/CDS | Fwd: 5'-TGGAATGAGGAGGACTGTAGAA-3'  Rev: 5'-CACCACCAGGTAGTTTGAAGAA-3'  Probe: 5'-/56-FAM/ATTGTACAT/ZEN/GAGCACCG GCTACCC/31ABkFQ/-3' | NM_007006 | 104 | 97.0 | 0.998 | -3.40 |
| huNUDT21/L-3’UTR | Fwd: 5’-CATTGCCAAAGAAGGCTTAGTG-3’  Rev: 5’-GAGTCCAGTTTAACCCAAATAATCAG-3’  Probe: 5'-/56-FAM/TGCTACAGA/ZEN/AAGCTGA CTTATACTACAGTC/31ABkFQ/-3' | NM_007006 | 127 | 106.8 | 0.993 | -3.17 |
| Hu18S-rRNA | Fwd: 5'-CTGAGAAACGGCTACCACATC-3'  Rev: 5'-GCCTCGAAAGAGTCCTGTATTG-3' | M10098.1 | 107 | 93.6 | 0.998 | -3.49 |

**Supplemental Table 2 List of antibodies used for western blot and immunocytochemistry in this study**

| Antibody | Catalog no. | Company | Species | Dilution | Solution | SDS-PAGE | Protein |
| --- | --- | --- | --- | --- | --- | --- | --- |
| Western Blotting | | | | | | | |
| β-actin | 013-24553 | WAKO | Mouse | 1:1000 | 3% skim milk | 12.5 % | 2 μg |
| CFIm25 | sc-81109 | Santa Cruz | Mouse | 1:1000 | 3% skim milk | 15 % | 10 μg |
| CFIm59 | sc-393880 | Santa Cruz | Mouse | 1:1000 | 3% skim milk | 10 % | 10 μg |
| CFIm68 | A301-356A | Benthyl Laboratories | Rabbit | 1:1000 | 3% skim milk | 10 % | 10 μg |
| eIFα | #5324 | Cell Signaling Technology | Rabbit | 1:1000 | 3% skim milk | 12.5 % | 10 μg |
| p-eIFα | #3398 | Cell Signaling Technology | Rabbit | 1:1000 | 3% skim milk | 12.5 % | 10 μg |
| Immunocytochemistry | | | | | | | |
| CFIm25 | SAB1404890 | Sigma | Mouse | 1:1000 | 2% blockace |  |  |
| CFIm68 | A301-356A | Benthyl Laboratories | Rabbit | 1:1000 | 2% blockace |  |  |
| PSPC1 | HPA038904 | Sigma | Rabbit | 1:1000 | 2% blockace |  |  |


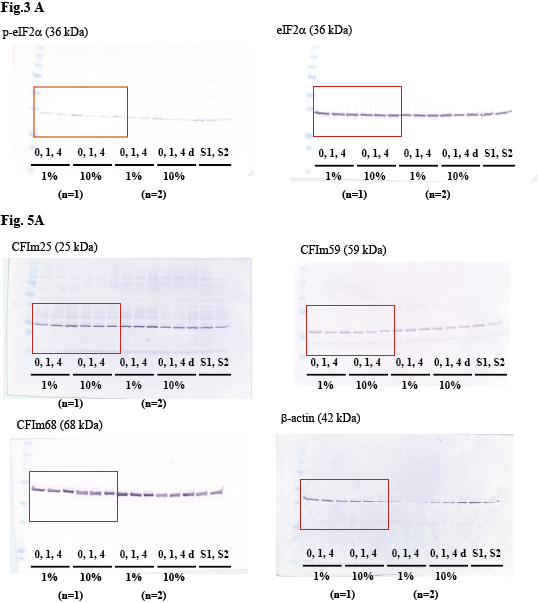


Supplemental Fig. S7 Raw data for Western Blotting
